# Adaptation and Molecular Characterization of Two Malaysian Very Virulent Infectious Bursal Disease Virus Isolates Adapted in BGM-70 Cell Line

**DOI:** 10.1155/2017/8359047

**Published:** 2017-11-05

**Authors:** Nafi'u Lawal, Mohd Hair-Bejo, Siti Suri Arshad, Abdul Rahman Omar, Aini Ideris

**Affiliations:** ^1^Department of Veterinary Pathology and Microbiology, Faculty of Veterinary Medicine, Universiti Putra Malaysia (UPM), 43400 Serdang, Selangor, Malaysia; ^2^Department of Veterinary Microbiology, Faculty of Veterinary Medicine, Usmanu Danfodiyo University, Sokoto (UDUS), 2346 Sokoto, Nigeria; ^3^Laboratory of Vaccine and Immunotherapeutics, Institute of Bioscience, Universiti Putra Malaysia (UPM), 43400 Serdang, Selangor, Malaysia; ^4^Department of Veterinary Clinical Studies, Faculty of Veterinary Medicine, Universiti Putra Malaysia (UPM), 43400 Serdang, Selangor, Malaysia

## Abstract

Two Malaysian very virulent infectious bursal disease virus (vvIBDV) strains UPM0081 and UPM190 (also known as UPMB00/81 and UPM04/190, respectively) isolated from local IBD outbreaks were serially passaged 12 times (EP12) in specific pathogen free (SPF) chicken embryonated eggs (CEE) by chorioallantoic membrane (CAM) route. The EP12 isolate was further adapted and serially propagated in BGM-70 cell line up to 20 passages (P20). Characteristic cytopathic effects (CPEs) were subtly observed at P1 in both isolates 72 hours postinoculation (pi). The CPE became prominent at P5 with cell rounding, cytoplasmic vacuoles, granulation, and detachment from flask starting from day 3 pi, up to 7 days pi with titers of 10^9.50^ TCID_50_/mL and log10^9.80^ TCID_50_/mL for UPM0081 and UPM190, respectively. The CPE became subtle at P17 and disappeared by P18 and P19 for UPM0081 and UPM190, respectively. However, the presence of IBDV was confirmed by immunoperoxidase, immunofluorescence, and RT-PCR techniques. Phylogenetic analysis showed that these two isolates were of the vvIBDV. It appears that a single mutation of UPM190 and UPM0081 IBDV isolates at D279N could facilitate vvIBDV strain adaptability in CEE and BGM-70 cultures.

## 1. Introduction

Infectious bursal disease (IBD) is a disease of high economic importance that is highly contagious and immunosuppressive affecting young chickens especially between 3 and 6 weeks of age. The etiologic agent, IBD virus (IBDV), is a naked icosahedral virus having segmented double stranded RNA genome belonging to the genus* Avibirnavirus*, family Birnaviridae [1]. The genome segment A encoding the viral polyprotein has been extensively studied using molecular techniques with the viral outer capsid protein (VP2) identified as the basis for antigenic and pathotypic variation among IBDV strains [2, 3]. It was also reported that specific amino acid changes in the hypervariable region of the VP2 protein determine the cell culture adaptation and attenuation of IBD virus [3]. The wild type IBD virus was reported to be extremely difficult to adapt to cell culture especially the very virulent IBDV (vvIBDV) unless passaged few times in chicken embryonated eggs (CEE) [4]. Traditional isolation of IBDV by CAM inoculation of 9- to 11-day-old SPF chicken embryos is expensive coupled with high risk of contamination especially for vaccine development and production [5]. Successful adaptation of IBDV isolates in chicken embryo fibroblast (CEF), chicken embryo kidneys, and chicken embryo bursas was reported [6, 7], but the cells being of primary avian origin have limited lifespan, produce low virus titer, and may contain extraneous avian viruses that may contaminate vaccines developed using them [8, 9]. Many continuous cell lines of mammalian origin were reported to support the growth of IBDV isolates including MA-104 [10], OK [11], BGM-70 [10, 12], Vero cells [10, 13–15], and RK-13 [14, 16]. These cell lines are easier to maintain and free from avian viral contaminants [9]. Various levels of viral titers were obtained using these cell lines making them better choices for IBDV propagation especially when higher viral titers are required as is the case in vaccine production. The objective of this study was to determine the adaptation and molecular characteristics of Malaysian vvIBDV isolates in BGM-70 cell line.

## 2. Materials and Methods

### 2.1. Viruses and Cell Line

The two local vvIBDV isolates were separately obtained from severe outbreaks of infectious bursal disease (IBD) in Malaysia in the years 2000 and 2004 and were designated UPM0081 (AY520910) [22] and UPM04/190 (AY791998) [23] also named as UPM190, respectively. These isolates were isolated from infected bursae that were ground, centrifuged, and filtered through a 0.2 *µ*m filter (Millipore, Merck). The filtrates were serially passaged via chorioallantoic membrane (CAM) inoculation of specific pathogen-free chicken eggs (SPF CEE) (MVP, Malaysia). After 7 days of incubation, the CAM were harvested, homogenized, and filtered and the CAM homogenate was stored at −80°C until required.

The cell line BGM-70 (ECACC cat number 90092601) is an epithelial-like cell derived from baby grivet monkey kidney (ECACC, Porton Down, Salisbury, SP4 0JG, UK) that was maintained in minimum essential medium (MEM) with 5% CO_2_ at 37°C.

### 2.2. Virus Adaptation in Specific Pathogen-Free Chicken Embryonated Eggs

To activate the vvIBDV isolates, 11-day-old SPF CEE were inoculated with the CAM homogenates of the two viruses via CAM route using an established method [24] and incubated at 37°C in an incubator. The CEE were observed daily for seven days for mortality discarding any dead embryo 48 hours postinoculation (pi). At 7 days pi, dead and surviving embryos were chilled at 4°C overnight and the CAM and embryos were aseptically harvested, homogenized with phosphate buffered saline (PBS, NaCl_2_-8 g/L, KH_2_PO_4_-0.2 g/L, NaH_2_PO_4_-1.15 g/L, KCl_2_-0.2 g, pH 7.2), and clarified at 1500 ×g for 20 minutes at 4°C. The supernatants for each isolate were pooled and 1% antibiotic and antifungal agents (penicillin 10,000 IU/mL, streptomycin 10,000 *µ*g/mL, and amphotericin B-25 *µ*g/mL) were added and filtered using 0.2 *µ*m filter and the filtrates were serially used for eleven more passages to give EP12 of both UPM0081 and UPM190 which were used for BGM-70 cell inoculation.

### 2.3. Adaptation of Viruses to BGM-70 Cell Line

Confluent monolayers of BGM-70 cells were infected with 500 *µ*L of EP12 vvIBDV at multiplicity of infection of 1 mean tissue culture infective dose (TCID_50_) of virus per cell following established method [25]. The viruses were adsorbed at 37°C for 2 hours with periodic gentle shaking of the flasks after which 5 ml of 2% MEM was added to each flask and were incubated at 37°C in 5% CO_2_ and examined twice daily for 7 days for development of cytopathic effects (CPE). The propagated viruses were harvested using three freeze-thaw cycles, following previously described method [26], filtered with 0.2 *µ*m (Millipore, Merck) aliquots, and labeled as BGMP1. This was serially repeated 19 times to obtain BGMP20. The TCID_50_ at BGMP5 was determined following standard method [27].

### 2.4. Detection of IBDV in BGM-70 Cell

The presence of vvIBDV in the cell culture supernatant was evaluated by apoptosis assay, immunofluorescence, immunoperoxidase [28], and reverse transcriptase-polymerase chain reaction (RT-PCR) [29].

### 2.5. Acridine Orange/Propidium Iodide Apoptosis Assay

The BGM-70 adapted viruses were evaluated for their ability to induce apoptosis in infected cells by double staining with acridine orange (AO) and propidium iodide (PI) DNA binding dyes [30]. Briefly, confluent monolayer of BGM-70 cells was infected with the adapted viruses as described before while another flask was kept as uninoculated control. The medium from infected flasks and control were separately collected 24 hrs pi and the monolayer was washed with warm PBS and trypsinized and the detached cells were aspirated into appropriately labeled tubes and centrifuged at 4°C at 1000 ×g. The supernatant was discarded while the pellets were resuspended with PBS and centrifuged again at 4°C at 1000 ×g and the supernatant was discarded, leaving only a small quantity of PBS to resuspend the cells. In a dark room, 5 *µ*L of AO dye stock solution (10 mg/mL, A3568, ThermoFisher Scientific) diluted in PBS (0.5 *µ*L of AO stock solution in 49.5 *µ*L of PBS) was mixed with 5 *µ*L of PI dye stock solution (1.0 mg/mL, P3566, ThermoFisher Scientific) diluted in PBS (5 *µ*L of PI stock solution in 45 *µ*L of PBS) and the mixture was added to 10 *µ*L of the infected cells or uninfected control, gently mixed on a glass slide, and immediately covered with cover slip. The slides were then viewed within 30 minutes with an immunofluorescence microscope (Leica Microsystems Limited, Heerbrugg, Switzerland).

### 2.6. Indirect Immunoperoxidase Assay

Four 6-well plates (Corning®, Sigma-Aldrich, Germany) containing confluent BGM-70 cells monolayer were taken out of the incubator and the medium was discarded and rinsed twice with prewarmed PBS, and the individual wells were inoculated with 200 *µ*L of the harvested cell culture supernatant of passages 5, 10, 15, 16, 17, 18, and 19 of both the UPM0081 and UPM190 and incubated for 120 minutes for adsorption at 37°C and 5% CO_2_. A volume of 1.8 mL of maintenance medium (MEM + 2% FBS) was added to each well, while the uninfected control wells were filled with 2 ml of the medium. The plates were then incubated at 37°C and 5% CO_2_ and observed daily for 4 days. The plates were fixed with 4% paraformaldehyde (P6148, Sigma-Aldrich, Germany) for 30 minutes at room temperature and washed twice using ice cold PBST (PBS containing 0.5% Tween 20) for 5 minutes and quenched with 3% hydrogen peroxide (H_2_O_2_) for 30 minutes at room temperature. The plates were washed twice with PBST for 5 minutes each and the VP2 IBDV antigen was retrieved with citrate buffer in a microwave (50 power level) for 10 minutes. The plates were rinsed with PBST twice for 5 minutes and blocked with 5% BSA in PBST for 1 hour at room temperature. The cells were rinsed twice as before and 40 *µ*l of monoclonal chicken anti-VP2-IBDV specific primary antibody (Charles River Laboratories, USA) diluted with distilled water at 1 : 200 was dispensed on the cells and then incubated at 4°C in humidified chamber overnight. The plates were rinsed two times with PBST for 5 minutes and 40 *µ*l of rabbit anti-chicken-IgY-Fc-HRP-conjugated secondary antibody (ThermoFisher Scientific) diluted with distilled water at 1 : 1000 was dispensed to each well and incubated at room temperature for 1 hour in a dark room. The plates were rinsed with PBST and 100 *µ*L/well of 3,3′ diaminobenzidine (Sigma-Aldrich, Germany) was added for 5 minutes of incubation, before the plates were briefly rinsed with PBST and stained with haematoxylin for 15 seconds followed by rinsing in slow running tap water for 5 minutes. The cover slips were dried and transferred to label clean glass slides using a mountant DPX (Sigma-Aldrich, Germany) and finally observed under the microscope (Leica Microsystems Limited, Heerbrugg, Switzerland) for positive reaction seen as brownish or golden coloration within the cytoplasm of the cells [28].

### 2.7. Indirect Immunofluorescence Assays

Clean coverslips were placed in four 6-well tissue culture plates and allowed to stand overnight under UV light exposure. The plates were seeded with BGM-70 cells following standard established protocols [26]. Following confluence, the plates were washed twice with prewarmed PBS, and the individual wells were inoculated with 200 *µ*l of the cell culture harvested supernatant of passages 5, 10, 15, 16, 17, 18, and 19 of both the UPM0081 and UPM190 viruses and incubated for 120 minutes for adsorption at 37°C, 5% CO_2_ condition, and then 1.8 mL of maintenance medium was added to each well, while the uninfected control wells were filled with 2 ml of the medium. The plates were then incubated at 37°C and 5% CO_2_ and observed daily for 3 days. The plates were fixed with 4% paraformaldehyde for 30 minutes at room temperature and washed three times for 5 minutes using ice cold PBST (PBS containing 0.5% Tween 20). After this, the plates were incubated in a 0.5% Triton X-100 in PBST (PBS 1 L pH 7.2 + 0.5 mL Tween 20) for 15 minutes to permeabilize the cells followed by rinsing with PBST three times for 5 minutes. Unspecific binding was blocked using blocking buffer (5% BSA in PBST) for 1 hour at room temperature. The plates were rinsed three times with PBST for 5 minutes and then 40 *µ*l of chicken monoclonal anti-VP2-IBDV specific primary antibody (Charles River Laboratories, USA) diluted at 1 : 200 with sterile distilled water was dropped on the cells followed by incubation at 4°C in a dark humidified chamber overnight. The plates were washed thrice for 5 minutes with PBST and 40 *µ*L of polyclonal rabbit anti-chicken-FITC conjugated secondary antibody raised against chicken IgY-Fc (Sigma*-*Aldrich, Germany) and diluted at 1 : 200 with sterile distilled water was dropped on each slide containing wells and incubated at room temperature for 1 hour in the dark. The plates were rinsed with PBST for 5 minutes three times and 20 *µ*l of 4′,6-diamidino-2-phenylindole dihydrochloride (DAPI) (Sigma-Aldrich, Germany) was added to the plates and incubated for 10 minutes at room temperature and the plates were briefly rinsed with PBST. The cover slips were dried and placed on a labeled clean glass slides using a mountant (DPX) for fluorescence microscopic examination (Leica Microsystems Limited, Heerbrugg, Switzerland) of VP2 antigen positive cells [28].

### 2.8. Reverse Transcriptase-Polymerase Chain Reaction (RT-PCR)

The CAM homogenate from CEE passage 12 and BGM-70 cell culture supernatants at passages 1, 5, 10, 15, 16, 17, 18, 19, and 20 were used for RNA extraction, cDNA synthesis, and PCR analysis. Briefly, 250 *µ*L of the CAM homogenate or cell culture supernatant was dispensed in 1.5 mL sterile Eppendorf tubes and 750 *µ*l of Trizol® LS was added in 1 : 3 ratio. The mixture was resuspended by several up- and downpipetting and allowed to stand for 15 minutes at room temperature. Chloroform (200 *µ*L) was added to the mixture, shaken vigorously for 15 seconds, and then allowed to stand at room temperature for 5 minutes before centrifugation at 12000 ×g for 15 minutes at 4°C. The upper clear aqueous phase containing RNA was gently removed from the two organic and DNA phases into new labeled 1.5 mL tube and was used for RNA precipitation. Five hundred microlitres of 100% isopropanol was added to each tube and allowed to stand at room temperature for 10 minutes before centrifugation at 12000 ×g for 10 minutes and the isopropanol was discarded while the RNA was washed with 1000 *µ*L of 75% alcohol and centrifuged for 5 minutes at 7500 ×g. The alcohol was discarded and RNA pellet partially dried inside level 2 biosafety cabinets for 5 to 10 minutes at the end of which 35 *µ*L of sterile RNAse free water was added to resuspend the RNA for determination of concentration, purity, and subsequent use for downstream application. The extracted RNA was used to amplify the VP2 hypervariable region of the segment A genomic RNA sequences.

### 2.9. cDNA Synthesis

The extracted RNA was used to synthesize cDNA using MMLV cDNA synthesis kit with the following reagent mixtures and conditions: RNA template (10 *µ*L), RNAase free water (1.5 *µ*L), and random oligomers (1.0 *µ*L). The mixture was briefly centrifuged and incubated at 65°C for 2 minutes and rapidly chilled on ice for 5 minutes after which 2.0 *µ*l of MMLV buffer, 2.0 *µ*L of DTT, 2.0 *µ*L of dNTPs, 0.5 *µ*L of Riboguard, and 1.0 *µ*L of reverse transcriptase were added to bring the total reaction volume to 20 *µ*L. The mixture was gently mixed, briefly centrifuged, and incubated at 37°C for 60 minutes after which the temperature was raised to 85°C for 5 minutes using the cyclical conditions shown in Table 1.

### 2.10. PCR Amplification

The synthesized cDNA was used as template for PCR amplification using KAPA HIFI PCR kit (Kapa Biosystems, Boston, Massachusetts, USA) using the following reagents volume and concentrations as recommended by the manufacturers: 5x KAPA HiFi Buffer 10.0 *µ*L (1x), 10 mM KAPA dNTP Mix 1.5 *µ*L (0.3 mM each), 10 *µ*M Forward Primer 1.5 *µ*L (0.3 *µ*M), 10 *µ*M Reverse Primer 1.5 *µ*L (0.3 *µ*M), Template DNA 5 *µ*L, 1 U/*µ*L KAPA HiFi DNA Polymerase 1.0 *µ*L (1 U), and 4.5 *µ*L PCR-grade water to top up to 25 *µ*L total reaction volume. The following primers described previously (Liu et al., 1994) were used to amplify a 643-bp region of the hvVP2 sequence: 643-1 (5′-TCACCGTCCTCAGCTTAC-3′) and 643-2 (5′-TCAGGATTTGGGATCAGC-3′). The mixture was briefly centrifuged and incubated in a PCR cycling conditions as shown in Table 2.

### 2.11. Nucleotide Sequence Analysis

To confirm the pathotypic identity of both the CEE and BGM-70 adapted viruses, all the amplified 643 bp PCR products from all the different passages were sequenced directly by Sanger method (First BASE Laboratories, Seri Kembangan, Selangor, Malaysia) and the nucleotide sequences were analyzed using BioEdit Sequence Alignment Editor v7.2.5 (Tom Hall, Ibis Biosciences, Carlsbad, CA). Phylogenetic analysis was conducted using the* MEGA* version 7 [19]. Alignment of nucleotide sequences was done using Clustal W, and Phylogenetic Trees were designed with the neighbor-joining (NJ) methods and up to 1000 bootstrap replicates. The portion of the sequence analyzed was from nucleotide positions 637 to 879 corresponding to the amino acid positions 213 to 293 with numbering according to [31]. The sequences used for comparisons comprised very virulent, variant, and classical serotype 1 as well as serotype 2 IBDV as shown in Table 3.

## 3. Results

### 3.1. Specific Pathogen-Free Embryonated Egg Passage

**Figure 1:**
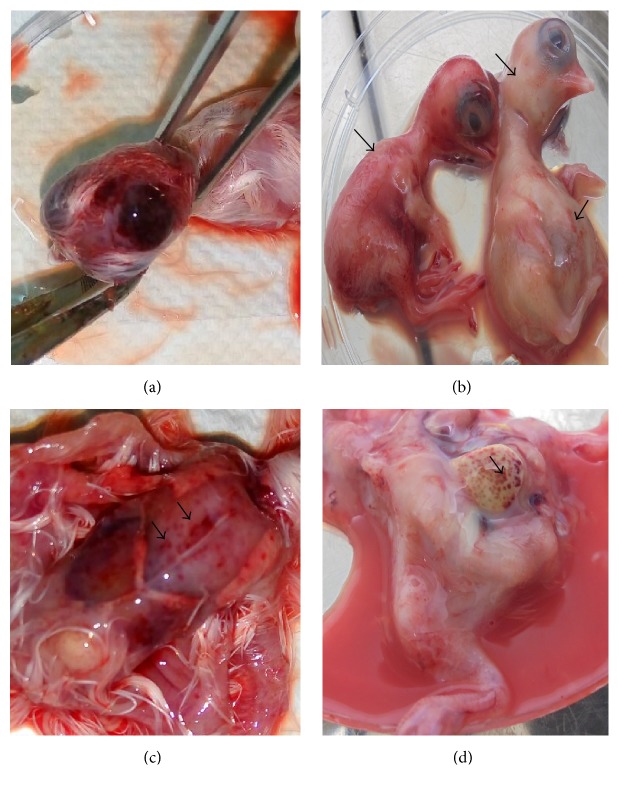
vvIBDV infected SPF embryos with (a) intracranial hemorrhage (UPM0081), (b) hyperemia, abdominal distention, and subcutaneous edema (UPM190), (c) petechial hemorrhages on the breast muscle (UPM0081), and (d) mottled liver (UPM0081) at EP12.

Both virus strains showed typical IBD lesions including intracranial hemorrhage, marbling of the liver, oedema of the head, hyperemia, and abdominal distention from EP2 to EP12. The mean embryo mortality was 5 days pi. The UPM0081 isolate presented lesions such as hyperemia, ecchymotic hemorrhages on the thigh, breast muscle, mottled liver (sometimes pale or yellowish), oedema of the head, intracranial hemorrhages, and dwarfing. The isolate UPM190 induced occasionally hyperemia or paleness, ecchymotic hemorrhages on the thigh and breast muscles, intracranial hemorrhages, distended abdomen, subcutaneous edema, and mottled liver (Figures 1(a)–1(d)) at EP12 of the two isolates.

### 3.2. Adaptation of Viruses to BGM-70 Cell Line

**Figure 2:**
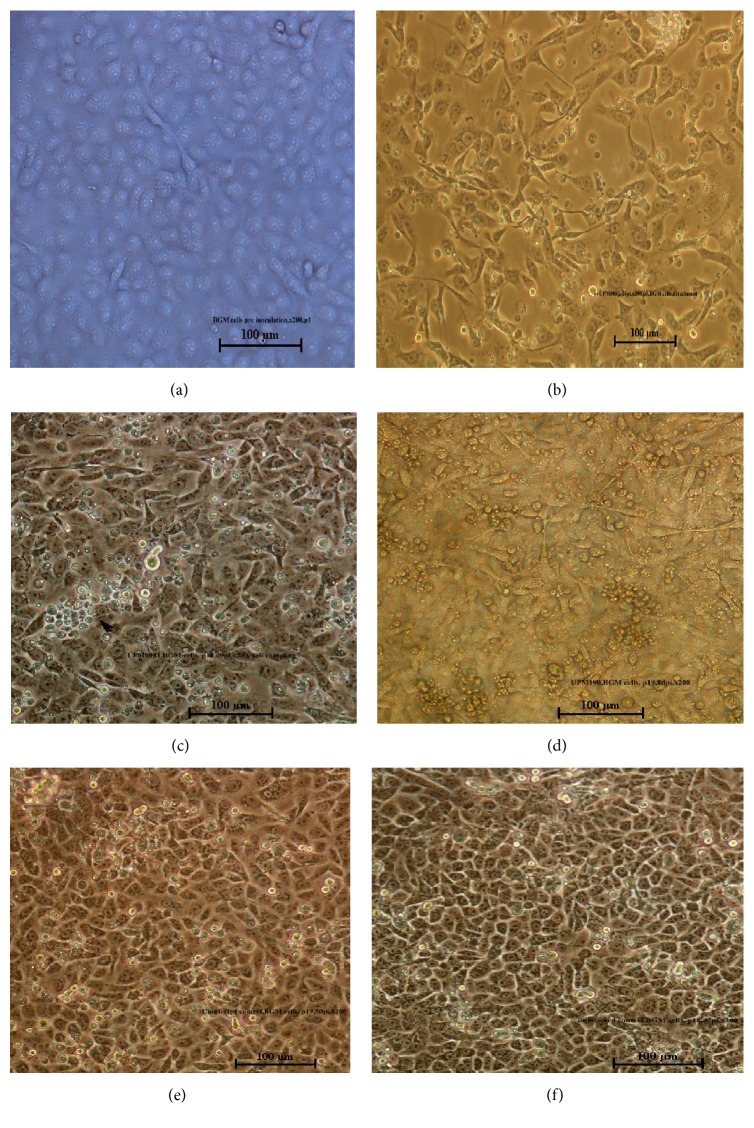
(a) A normal confluent monolayer of BGM-70 cells compared with (b) UPM0081, BGMP5 induced CPE on BGM-70 infected cells including small refractive cells, cytoplasmic granulation, cell rounding, and detachment at 6 days pi. (c) BGM-70 cells infected with UPM0081 at BGMP18 and (d) UPM190 BGMP19 at days 9 and 8, respectively, showing little CPE compared with (e) uninfected controls at day 5 and (f) at day 7. Bar = 100 *µ*m.

Normal confluent monolayer of BGM-70 cells was obtained within 72 hours with fibroblast morphology (Figure 2(a)). During the first passage, the virus induced little CPE with the monolayer remaining 100% intact up to 4 days pi. Evidence of CPE manifested from day 5 pi as the virus began adapting to the cell line. At BGMP2, CPE developed 4 days pi and reached 20% by day 7 pi and by P5; clear CPE developed within 48 hours pi. The CPE was characterized by small refractile and round cells, cytoplasmic granulation, cell detachment, and slow destruction of monolayer by day 6 pi (Figure 2(b)). The CPE became subtle at P18 onwards appearing only after 9 days pi suggesting reduction in virulence of the propagated viruses (Figures 2(c) and 2(d)). The infectious titer of the adapted viruses at BGMP5 was found to be 10^9.98 ^TCID_50_/mL and 10^9.50 ^TCID_50_/mL at 3 days pi for UPM190 and UPM0081, respectively.

### 3.3. Acridine Orange/Propidium Iodide Apoptosis Assay

**Figure 3:**
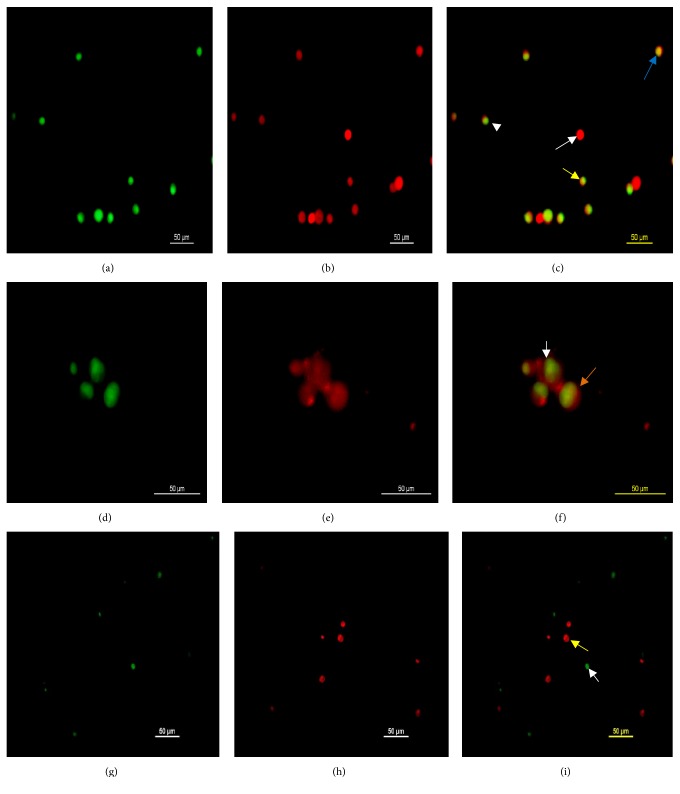
(a) AO stained, (b) PI stained, and (c) merged UPM0081 infected BGM-70 cells showing green-orange fluorescence as an indicator of early (white arrowhead), intermediate (yellow arrow), and late apoptosis (blue arrow) and necrosis (white arrow) at 24 hours pi. Bar = 50 *µ*m. (d) AO stained, (e) PI stained, and (f) merged UPM190 infected BGM-70 cells showing membrane blebbing (white arrow) and positive orange color induced by the virus at 48 hours pi as a sign of apoptosis. Bar = 50 *µ*m. (g) AO stained, (h) PI stained, and (i) merged uninfected control BGM-70 cells showing normal unapoptotic (white arrow) and necrotic cells (yellow arrow) at 24 hours after culture. Bar = 50 *µ*m.

The apoptosis assay using AO/PI dyes showed that, at 24 hours pi, the IBDV triggered few infected cells to undergo apoptosis as a means of virus dissemination (Figure 5). The AO dye stains the nucleus of both normal and apoptotic cells green as it became bound to DNA because it can easily penetrate intact membrane of living cells, whereas PI dye can only permeate cells that have damage membrane such as apoptotic and necrotic cells which it stains orange-red to red. Apoptotic cells were demonstrated by the presence of orange coloration (Figures 3(c) and 3(f)) due to the combined effects of the AO/PI dyes and membrane blebbing (Figure 3(f)) at 24 and 48 hours pi compared to the uninfected control (Figure 3(i)).

### 3.4. Indirect Immunoperoxidase Assay

**Figure 4:**
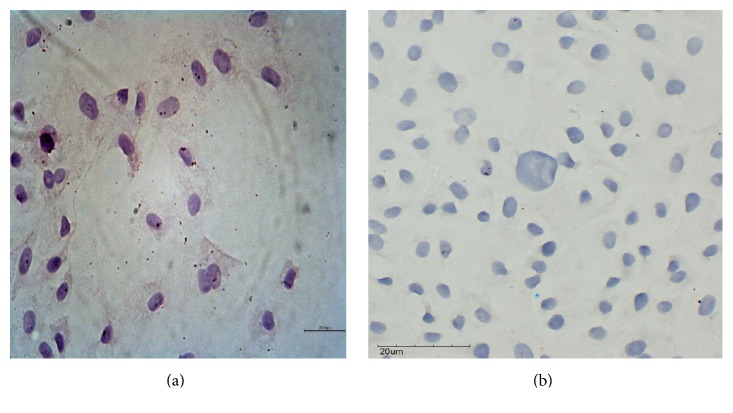
(a) Immunoperoxidase positive BGM-70 cells showing brown cytoplasmic precipitate compared to (b) the uninfected BGM-70 cells.

**Figure 5:**
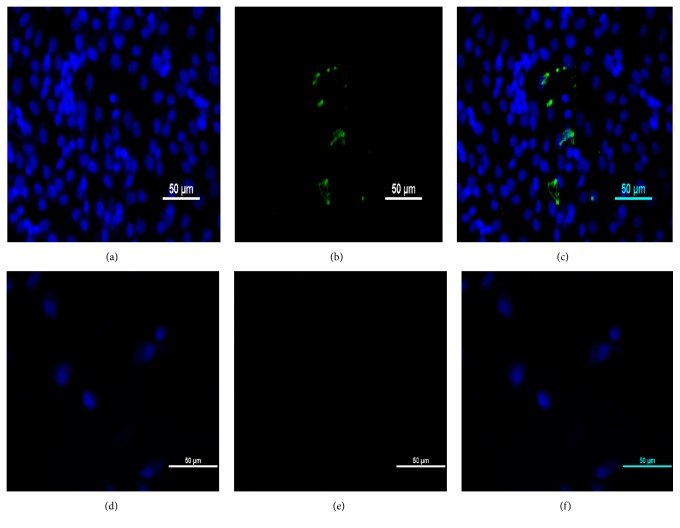
IBDV infected BGM-70 cells showing (a) blue fluorescence when stained with DAPI (b) green fluorescence when stained with FITC-labeled anti-chicken antibody indicating the presence of VP2 antigen and (c) the fluorescence when the two channels were merged (c). The uninfected BGM-70 control (d, e, and f) showing no green fluorescence due to the absence of IBDV VP2 antigen within the cells.

To verify the presence of IBD viruses in infected BGM-70 cells, indirect immunoperoxidase assay was performed on infected cells grown on glass slides. The assay resulted in the presence of intracytoplasmic brown coloration that indicates the presence of the IBDV VP2 antigen in the cytoplasm of BGM-70 infected cells (Figure 4(a)) compared to the uninfected control (Figure 4(b)).

### 3.5. Indirect Immunofluorescence Assay

To further confirm the presence of the VP2 viral antigen within the cytoplasm of BGM-70 cells, indirect immunofluorescence assay was performed. The assay revealed positive green fluorescence for IBDV VP2 protein in the cytoplasm and on the nuclear membrane of infected cells (green) compared to the uninoculated negative control that showed only excitation for the nuclear stain DAPI (blue). The presence of the green signal for VP2 antigen therefore indicates the presence of IBD virus in the cytoplasm and on the nuclear membrane of infected BGM-70 cells.

### 3.6. Reverse Transcriptase-Polymerase Chain Reaction (RT-PCR)

**Figure 6:**
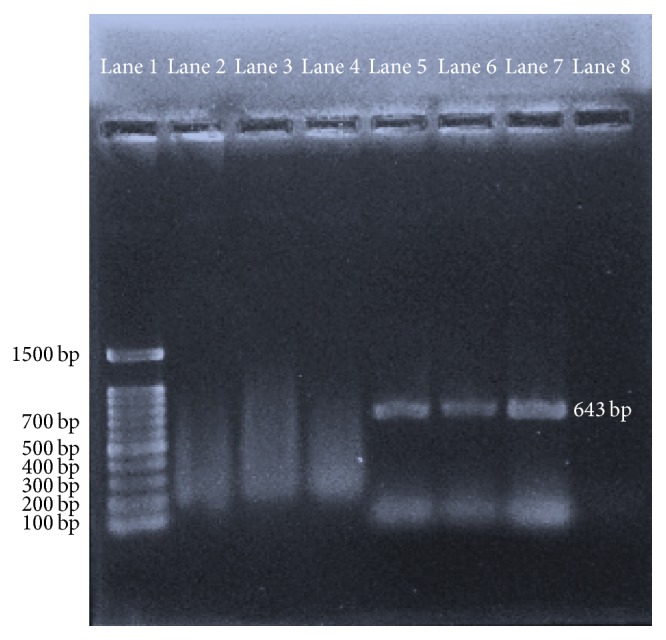
RT-PCR products of the BGM-70 cell culture supernatants showing 100 bp molecular ladder (Lane 1), nontemplate control (Lanes 2 and 3), negative control (Lane 4), positive control (Lane 5), UPM0081 (Lane 6), and UPM190 (Lane 7).

The identification of CEE and BGM-70 adapted viruses through embryonic lesions and CPE was confirmed by the appearance of a distinct 643 bp band when an RT-PCR of the CAM homogenate and BGM-70 cell culture supernatant and the products were analyzed by gel electrophoresis and ethidium bromide staining (Figure 6).

### 3.7. Nucleotide Sequence Analysis

**Figure 7:**
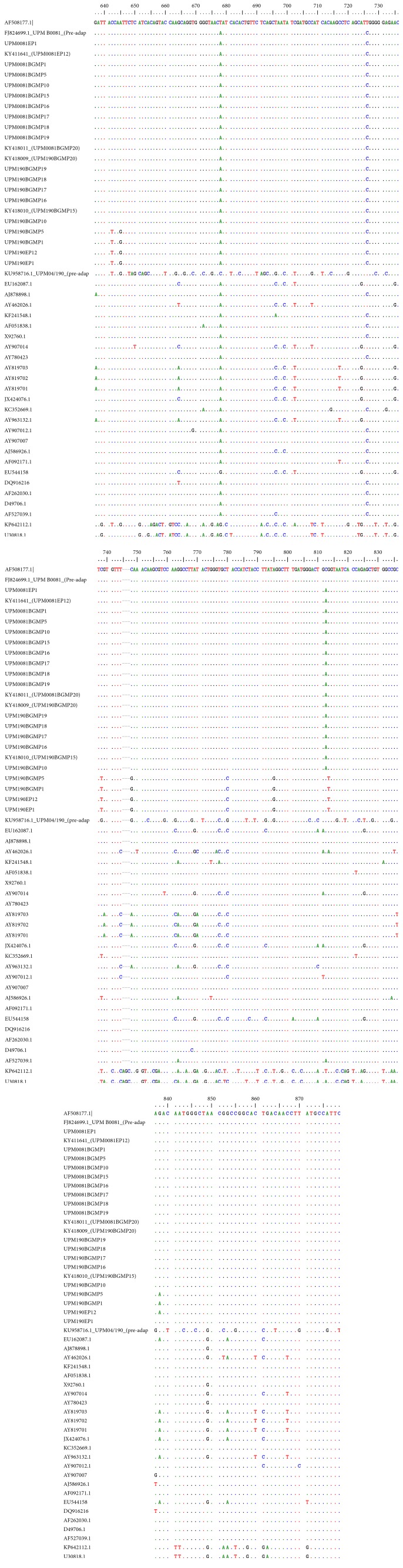
Nucleotide sequences of the isolates. Nucleotide sequences of 645 bp fragment of the hypervariable region of VP2 comparison between CEE and BGM-70 adapted Malaysian isolates and reference sequences. Dots (.) indicate consensus with the AF508177.1, a South Korean IBDV isolate, and dash (-) indicates gaps.

To confirm the identity of the RT-PCR products as IBDV genome, the RT-PCR products were directly sequenced and analyzed using BioEdit* v*7 and MEGA* v*7. The nucleotide sequences of the two isolates were aligned by ClustalW together with other reference sequences (Figure 7). The aligned sequences revealed that, at nucleotide positions 642 and 645, there were changes from C and A to T and G nucleotides in the sequences of preadaptation UPM04/190 parent virus, UPM190EP1, UPM190EP12, UPM190BGMP5, and UPM190BGMP10. From positions 648 to 650, UPM04/190 had TAG compared to the CTC possessed by the rest of the isolates including the reference sequences. At nucleotide positions 652 to 654, there were nucleotide mutations from TCA to AGC in UPM04/190. Other nucleotide mutations observed were C660T, A663G, A666G, T669C, A675G, A693C, T696C, T699C, C702T, C708G, C711T, A714C, C720G, C726T, G729C, A732C, A752C, C759G, T758G, T777C, and C780G. Others include C786T, C789T, T792G, G807C, T810C, A816G, C819T, A823C, and A825T. Further changes were detected at T828G, C834G, A837G, C840T, T843C, G846C, A849G, G852C, C855G, T861C, C864T, T870G, A876G, and C879T. Moreover, the isolates UPM190EP1, UPM190EP12, UPMBGMP5, and UPMBGMP10 nucleotide changes were observed in some reference sequences.

**Figure 8:**
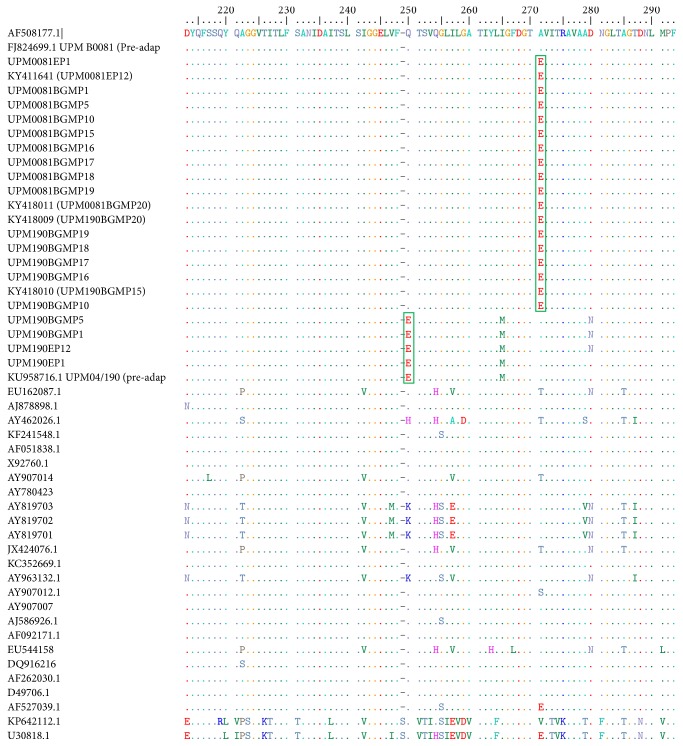
Deduced amino acid sequences of the isolates. Figure 8 shows the deduced amino acid sequences from positions 213 to 293 where both CEE and BGM-70 adapted UPM190 and UPM0081 isolates showed high similarity with the reference vvIBDV from different parts of the world such as UK661, HK46, JNeto-BR, SA-KZN95, Oyo.NIE 96-09, IRAQ12.127-743 but striking differences with the classical, variant, and serotype 2 reference isolates. Note the E249 and E270 (boxed) amino acids present in some of adapted viruses not seen in other vvIBDV except UPM94/273, a Malaysian isolate with unusual pathogenicity and serotype 2 OH strain.

When the nucleotide sequences of the preadaptation, CEE, BGM-70 adapted, and reference isolates were translated to amino acids, the putative motifs identifying vvIBDV pathotypes were seen revealing isoleucine (I) at positions 242, 256, and 294 and serine (S) at position 299 (Figure 8). The IDA motifs at amino acid positions 234 to 236 were present in both the preadaptation and CEE and BGM-70 adapted viruses (Figure 8). At position 249, UPM04/190, UPM190EP1, UPM190EP12, UPM190BGMP1, and UPM190BGMP5 possessed amino acid E249 in place of Q249 possessed by the rest of the CEE and BGM-70 adapted strains. Similarly, all the reference sequences possessed Q249 except for KF241548.1 that possessed H249. Similarly, at position 270, UPM B0081 preadaptation virus, UPM04/190, UPM190EP1 and UPM190EP12, UPM190BGMP5 and UPM190BGMP10, and other reference sequences possessed Alanine at that position while the other CEE and BGM-70 adapted viruses had glutamic acid (E270) at that position. The reference sequences however possessed A, T, S, or V in that position except AF527039.1 and U30818.1 which possessed the E270 amino acid. At amino acid position 279, only UPM190EP12, UPMBGM190P1, and UPMBGM190P5 possessed an Asparagine (N) while the preadaptation parent virus and other CEE and BGM-70 adapted isolates possessed D (Figure 8). The N279 mutation however reverted back as the viruses were further serially passaged in the BGM-70 cell line.

**Figure 9:**
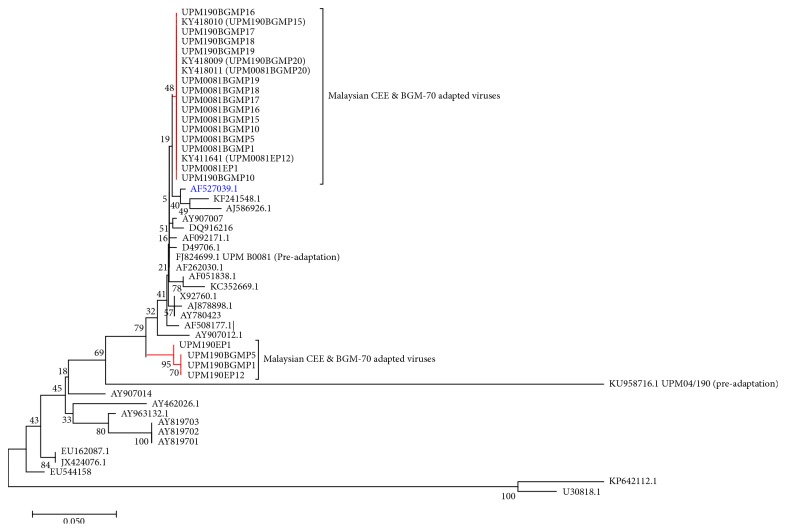
Nucleotide Phylogenetic Tree Analysis. Phylogenetic analysis of the hypervariable region of the VP2 gene of the IBDV segment A genome from nucleotide positions 637 to 876. The preadaptation, CEE, and BGM-70 adapted viruses are located on the red branch. The vvIBDV pathotypes included in the analysis were AJ878898, AF051838, KF241548, AF508177, D49706.1, X92760, AY780423, AF262030.1, AY520910.1, AF092171.1, AY907007, KC352669.1, AY907012.1, AJ586926.1, and DQ916216. JX424079.1, AY462026, EU162087, AY819703, EU544158, AY907014, AY819702, AY819701, and AY963132.1 represent the classical strains, whereas U30818.1 and KP642112.1 represent the serotype 2 sequences. The evolutionary history and distances were inferred using the neighbor-joining method [17] and the Kimura 2-parameter method [18], respectively. Evolutionary analyses were conducted in MEGA7 [19].

**Figure 10:**
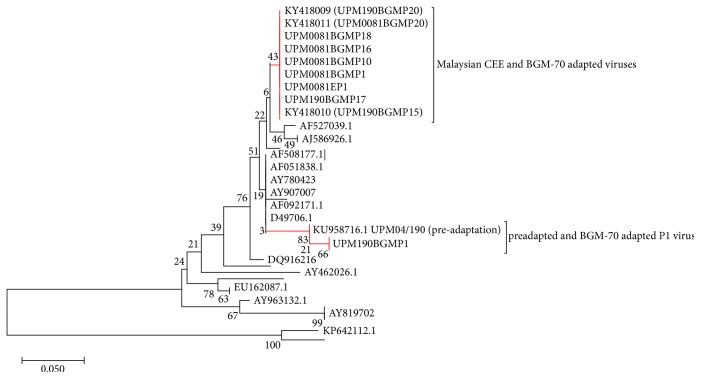
Phylogenetic Tree of the deduced amino acids. Fifty amino acid sequences were used to infer evolutionary history using the neighbor-joining method [17] and 1000 bootstrap test [20]. The Poisson correction method was used to compute the evolutionary distances [21]. Analyses were conducted in MEGA7 [19].

Phylogenetic analysis of the nucleotide sequences revealed that our isolates cluster with the sequences of the vvIBDV isolates from Europe, Asia, Middle East, and Africa deposited in GenBank included in the analysis (Figure 9). Distance matrix (Figure 10) revealed pairwise similarity index ranging from UPM B0081 was 0.8% different with AF508177, 10.9% different with the cell culture adapted Edgar strain, 11.5% different with AY819701, 9% different with AY963132, 8% different with JX424076.1, 43.4% different with U30818, and 45.9% different with KP642112.1. When compared with the UPM190 and UPM0081 sequences, the preadaptation UPM B0081 was 33.8% different with UPM04/190 and 0.4% to 3.4% different with the rest of the CEE and BGM-70 adapted isolates. On the other hand, UPM04/190 was 33.2% different with AF508177 and 34.5% different with all the CEE and BGM-70 except UPM190BGMP1 and UPM190BGMP5 and UPM190EP12 that differs by 32.5% and 31.8% with UPM190P1. Moreover, when UPM04/190 was compared with the serotype 2 isolates, U30818.1 was 62.9% different with it while KP642112 differs with it by 64.9%. The rest of the CEE and BGM-70 adapted isolates differed with UPM04/190 between 0.4% and 3.9%. The sequences used to construct the Phylogenetic Tree and to compute distance matrix are shown in Table 3.

## 4. Discussion

Outbreaks of vvIBDV in chickens as confirmed through RT-PCR and restriction fragment length polymorphism (RFLP) techniques were reported in Malaysia [22] and other parts of the world. Primary SPF CEE and several continuous avian and mammalian cell lines including BGM-70 cells were reported to support the growth and propagation of many viruses infecting vertebrates and invertebrate species including IBDV [9, 10, 12, 21]. In this study, two Malaysian vvIBDV isolates designated UPM0081 and UPM190 were adapted and serially passaged 12 times in CEE and were later adapted to grow in BGM-70, a continuous cell line of mammalian origin. The advantages of continuous mammalian cell lines over CEE include among other things such as absence of contamination with avian viruses, ease of handling, low cost, infinite lifespan, and ease of maintenance [9]. Most of the commercially available vaccines for the control of IBDV infection especially the very virulent IBDV (vvIBDV) pathotype are egg based, making their production laborious, costly, and a possible source of vertical transmission for important avian viruses to the vaccinated flocks. The need for shift from egg based to cell culture based IBD vaccine development using vvIBDV as the seed virus is very crucial in order to reduce the labor and high cost of production involved, but the problem is that vvIBDV has been reported to be difficult to adapt to cell culture [32, 33]. The unsuccessful attempt to adapt vvIBDV isolates from Holland, Taiwan, and Turkey on BGM-70 cell line after 10 blind passages has been previously documented [25]. In that study, the vvIBDV strains were passaged 8 times in SPF CEE before using it for serial inoculation of BGM-70 cultures up to 10 times without CPE development. In this study, two Malaysian vvIBDV isolates UPM0081 and UPM190 were successfully adapted on BGM-70 cell line with distinctive CPE development by passage 5. This is similar to the observation of Hassan and coworkers [9] and Abdel-Alim and Saif [34] where classical (STC) and variant (IN) IBDV were successfully adapted on BGM-70 cells within two and three blind passages, respectively, with characteristic CPE development. Furthermore, the reports of El-mahdy et al. [12] on the adaptation of local Egyptian vaIBDV isolates on BGM-70 cell line at passage 1 and with high virus yield (log_10_⁡7.5  TCID_50_/mL) agreed with our findings as IBDV UPM190 and UPM0081 yielded high virus titer of 10^9.98 ^TCID_50_/mL and 10^9.50 ^TCID_50_/mL, respectively, 72 hours after infection at passage 5.

Earlier investigations on the usefulness of BGM-70 cell line for the propagation of IBDV indicated that serial propagation of classic (STC) and variant (IN) IBDV as low as 4 passages resulted in loss of pathogenicity [9]; similarly, serial passaging as high as 30 passages (variant IN strain) or 40 passages (variant E strain) leads to the loss of ability of the viruses to replicate in the bursa of SPF chickens when used as live vaccines but protects chickens against experimental challenge when the viruses were used as inactivated vaccines [26]. In this study, the two viruses were passaged 20 times only in BGM-70 cells with high virus titer yield (log10 9.98 TCID_50_/mL and log10 9.50 TCID_50_/mL for UPM190 and UPM0081, respectively); there is the need to evaluate their pathogenicity, immunogenicity, and efficacy in experimental challenge studies to establish their usefulness as potential live attenuated or inactivated vaccine candidates.

Apoptosis is a programmed cell death that is highly regulated by cellular factors characterized by distinct morphological cytologic changes such as chromatin condensation, nuclear fragmentation, and membrane blebbing [35]. Certain staining methods were developed that aid in the recognition and differentiation of apoptotic from necrotic cells which include Annexin V stain, 4′,6-diamidino-2-phenylindole (DAPI) stain, acridine orange/ethidium bromide/propidium iodide stain, and Hoechst stain. These fluorochromes emit fluorescence when they bind to DNA and are viewed at certain excitation spectra with fluorescence microscopy [36]. The productive infection of cell lines with IBDV has been associated with induction of apoptosis [30, 37, 38], a process that was linked to the nonstructural IBDV VP5 protein, seen only in IBDV infected cells [37, 39–41]. The use of AO/PI dyes to stain infected cultures was used to differentiate apoptotic from necrotic cells [30, 36]. The presence of an orange coloration after dual staining with AO/PI was considered as one of the indicators of apoptosis beside nuclear fragmentation, condensation, and membrane blebbing [42]. In this study, AO/PI dyes were used to demonstrate apoptosis induced by the VP5 nonstructural protein of IBDV that is seen only in a productive infection [30, 37–41, 43, 44]. The dual staining of IBDV infected BGM-70 cells with AO/PI dyes allowed the detection of cells undergoing apoptosis evidenced by the presence of orange coloration of the nucleus of stained cells when viewed under fluorescence microscopy. Presence of propidium iodide in a cell indicates loss of cell membrane integrity; therefore this dye can only permeate damaged cells where it stains the nucleus red. Acridine orange on the other hand readily crosses the membrane of healthy and early apoptotic cells and stains the nucleus green. The presence of these two dyes together in a cell staining the nucleus orange to red is an evidence of the loss of membrane selective permeability that is only seen in dying cells. Staining of the nucleus red by the PI in combination of AO indicates necrotic cells whereas orange coloration is an indication of cells undergoing late apoptosis [42]. This technique is simple but elegant in its ability to differentiate dead cells from those undergoing apoptosis in response to obnoxious stimuli such as IBD virus.

To further confirm the presence of IBDV within infected BGM-70 cells, immunohistochemistry and immunofluorescence techniques were performed to identify antigen positive cells [44–48]. The presence of VP2 antigen as demonstrated by immunoperoxidase and immunofluorescence within the cytoplasm of infected cells 48 hours pi indicated the ability of BGM-70 cell line to support the growth and replication of these two vvIBDV isolates, because the virus was reported to replicate within the cytoplasm of infected cells with generation of viral capsid proteins (VP2 and VP3) within 48 hours pi [30, 49, 50]. These two techniques are sensitive and specific and useful diagnostic tools for the detection and localization of IBDV in infected cells and tissues.

The molecular analyses using RT-PCR, sequence, and sequence analysis revealed that the two isolates are phylogenetically related to the vvIBDV found in Asia, Europe, Middle East, and Africa due to the presence of A222 [51, 52] and I242, I256, I294, and S299 [51] in their VP2 hypervariable amino acid composition compared to the caIBDV or vaIBDV that possessed V222, VA, or E256 or the serotype 2 viruses that possessed P222, V242, I or S256, N or F294 and T or P299 [51] in the same region. The presence of the 233–236 IDA motif in our strains just like other IBDV indicates that these viruses could adapt to cell culture because these amino acids were reported to be important for cell culture adaptation [51, 53] via the integrin receptors. Furthermore, during the CEE passaging, UPM190 isolate acquired a mutation at D279N at EP12, a mutation that was reported to be part of the two changes observed in attenuated and/or cell culture adapted IBD viruses [7, 54–56]. However, when the virus was passaged in BGM-70 cell, the N279 reverted back to D279 as seen in the amino acid sequences of the BGM-70 adapted viruses. The ability of this isolate with only a single mutation at D279N to replicate in BGM-70 cell culture indicated that perhaps the D279N mutation alone could facilitate cell culture adaptability to a very virulent strain of IBDV. This may be the reason why UPM190 replicated more efficiently and with more pronounced CPE compared to UPM0081 whose amino acids remained unchanged during the CEE passages. Moreover, BGM-70 adaptation resulted in other mutations in UPM190 amino acid sequences at positions E249Q and A270E within the VP2 hypervariable region. The A270E mutation was previously reported only in UPM94/273, a vvIBDV with unusual pathogenicity, and in nonpathogenic serotype 2 OH strain [57]. It was previously reported that two amino acid changes at specific sites may lead to virus attenuation [58]. The appearance of this A270E mutation in the sequence of BGM-70 adapted UPM190 may be responsible for the decrease in CPE observed in BGM-70 cell culture suggesting attenuation. However, for UPM0081, the A270E mutation was acquired during CEE adaptation before the isolate was passaged and adapted in BGM-70 cell line. The importance of this mutation needs to be confirmed by further experiments involving reverse genetic technology.

Phylogenetically, all the isolates fall within the vvIBDV clades by clustering with other published vvIBDV reference sequences deposited in GeneBank, indicating that the BGM-70 passaging and the mutations observed did not lead to a change in the pathotypes of the isolates.

In summary, attenuated Malaysian vvIBDV was obtained by 12 serial passages in CEE followed by 20 serial passages in BGM-70 cell line. One amino acid change was seen in UPM190 at CEE passage 12 that reverted back when the isolate was passaged in BGM-70 cell line, and two amino acid changes were seen in UPM190 and one mutation was seen in UPM0081 during BGM-70 serial passages. To our knowledge, this is the first reported successful adaptation of local Malaysian vvIBDV isolates in BGM-70 cell line; therefore, this study highlight the merit of this cell line for the* in vitro* propagation of vvIBDV in the laboratory and the possible role the A270E mutation may play as a novel site for attenuation, further strengthening the possibility that many different sites within the VP2 hypervariable region may be involved in IBDV attenuation.

## Figures and Tables

**Table 1:** cDNA synthesis using IBDV RNA templates.

Condition	Temperature (°C)	Time	Cycle
First denaturation	98	2 min	1
Second denaturation	98	30 sec	35
Annealing	56	30 sec	35
Extension	72	1 min	35
Final extension	72	10 min	1

**Table 2:** PCR cycling conditions used to amplify the synthesized cDNA templates.

Condition	Temperature	Time	Cycle
Initial denaturation	95°C	3 min	1
Denaturation	98°C	20 sec	35
Annealing	60°C	15 sec	35
Extension	72°C	15–60 sec/kb	35
Final extension	72°C	1 min/kb	1

**Table 3:** The names, accession number, countries of origin, and references of the sequences used for nucleotide, amino acid, and phylogenetic analyses with the UPM0081 and UPM190 isolates.

S/number	Sequence	Strain	Accession number	Country	References
(1)	UPM94/230	vvIBDV	AY520911.1	Malaysia	Tan et al., 2004
(2)	UPM94/273	vvIBDV	AF527039.1	Malaysia	Kong et al., 2004
(3)	UPM B0081	vvIBDV	FJ824699.1	Malaysia	Mohammed et al., 2009
(4)	UPM92-04	vvIBDV	AF262030.1	Malaysia	
(5)	Strain Harbin	vvIBDV	AF092171.1	China	Hu & Zhang, 1998
(6)	Strain SA-KZN95	vvIBDV	KF241548.1	South Africa	Vukea et al., 2014
(7)	IBDV77/Georgia Vac	Vaccine	JX424076.1	Nigeria	Adamu, 2012
(8)	Strain Edgar	cell culture adapted	AY462026.1	USA	Petkov et al., 2007
(9)	South Korea (Strain KSH)	vvIBDV	AF165151.1	South Korea	Kwon et al., 2000
(10)	UK661	vvIBDV	X92760.1	UK	Brown and Skinner, 1996
(11)	GA97	vIBDV	AY963132.1	USA	Mickael and Jackwood, 2005
(12)	IRAQ12.I27-743	IBDV	KC352669.1	Kurdistan and Iraq	Gergis and Jackwood, 2013
(13)	ISR13	vvIBDV	AY907012.1	USA	Jackwood and Sommer, 2005
(14)	UPM0081EP12	vvIBDV	KY411641	Malaysia	Lawal, Hair-Bejo, Arshad, et al., 2016
(15)	UPM190EP12	vvIBDV		Malaysia	Lawal, Hair-Bejo, Arshad, et al., 2016

S/number	Name	Strain	Accession number	Country	References

(16)	IBDV/Turkey/PA/00924/14	Serotype 2	KP642112.1	USA	Lu, Tang, Yeh, et al., 2015
(17)	OKYM	vvIBDV	D49706.1	Japan	Yamaguchi, Ogawa, Inoshima, et al., 1995
(18)	Thai4 classic	caIBDV	AY907014	Thailand	Jackwood and Sommer, 2005
(19)	JNeto-BR	IBDV	AY780423	Brazil	Hayashi, Brentano and Ferreira, 2004
(20)	Strain E	vIBDV	AY819703	USA	Khatri and Sharma, 2004
(21)	Strain IM	vIBDV	AY819702	USA	Khatri and Sharma, 2004
(22)	Strain STC	caIBDV	AY819701	USA	Khatri and Sharma, 2004
(23)	Spain 1	vvIBDV	AY907007	Spain	Jackwood and Sommer, 2005
(24)	D78	vvIBDV	EU162087.1	USA	Jackwood, D. J., Sreedevi, LeFever et al., 2008
(25)	Strain B00/81 (Pre-adaptation)	vvIBDV	AY520910.1	Malaysia	Tan et al., 2004
(26)	HK46	vvIBDV	AF092943	Hong Kong	Lim, Cao, Yu et al., 1999
(27)	Cevac-Gumbo-L	Vaccine	EU544158	Brazil	Gomes, Abreu, Resende et al., 2008
(28)	Singapore97S181	IBDV	DQ916216	Singapore	Jackwood and Sommer-Wagner, 2006
(29)	OH strain	Serotype 2	U30818.1	Canada	Kibenge, McKenna and Dybing, 1995
(30)	UPM190BGMP15	vvIBDV	KY418010	Malaysia	Lawal, Hair-Bejo, Arshad, et al., 2016
(31)	UPM190BGMP20	vvIBDV	KY418009	Malaysia	Lawal, Hair-Bejo, Arshad, et al., 2016
(32)	UPM0081BGMP20	vvIBDV	KY418011	Malaysia	Lawal, Hair-Bejo, Arshad, et al., 2016
(33)	UPM04/190 (Pre-adaptation)	vvIBDV	KU958716.1	Malaysia	Liew, Hair-Bejo, Omar et al., 2016
(34)	UPM190EP1	vvIBDV		Malaysia	Lawal, Hair-Bejo, Arshad, et al., 2016
(35)	UPMBGMP0081P15	vvIBDV	KY418012	Malaysia	Lawal, Hair-Bejo, Arshad, et al., 2016
(36)	UPMBGMP0081P16	vvIBDV		Malaysia	Lawal, Hair-Bejo, Arshad, et al., 2016
(37)	UPMBGMP0081P17	vvIBDV		Malaysia	Lawal, Hair-Bejo, Arshad, et al., 2016
(38)	UPMBGMP0081P18	vvIBDV		Malaysia	Lawal, Hair-Bejo, Arshad, et al., 2016
(39)	UPMBGMP0081P19	vvIBDV		Malaysia	Lawal, Hair-Bejo, Arshad, et al., 2016
(40)	UPMBGMP0081P10	vvIBDV		Malaysia	Lawal, Hair-Bejo, Arshad, et al., 2016
(41)	UPMBGMP0081P5	vvIBDV		Malaysia	Lawal, Hair-Bejo, Arshad, et al., 2016
(42)	UPMBGMP0081P1	vvIBDV		Malaysia	Lawal, Hair-Bejo, Arshad, et al., 2016
(43)	UPMBGMP190P16	vvIBDV		Malaysia	Lawal, Hair-Bejo, Arshad, et al., 2016
(44)	UPMBGMP190P17	vvIBDV		Malaysia	Lawal, Hair-Bejo, Arshad, et al., 2016
(45)	UPMBGMP190P18	vvIBDV		Malaysia	Lawal, Hair-Bejo, Arshad, et al., 2016
(46)	UPMBGMP190P19	vvIBDV		Malaysia	Lawal, Hair-Bejo, Arshad, et al., 2016
(47)	UPMBGMP190P10	vvIBDV		Malaysia	Lawal, Hair-Bejo, Arshad, et al., 2016
(48)	UPMBGPM190P5	vvIBDV		Malaysia	Lawal, Hair-Bejo, Arshad, et al., 2016
(49)	UPMBGMP190P1	vvIBDV		Malaysia	Lawal, Hair-Bejo, Arshad, et al., 2016
(50)	UPM190EP1	vvIBDV		Malaysia	Lawal, Hair-Bejo, Arshad, et al., 2016
(51)	South Korean IBDV	vvIBDV	AF508177.1	South Korea	Kim and Yeo, 2002
